# A Comprehensive Review of Clinical Cardiotoxicity Incidence of FDA-Approved Small-Molecule Kinase Inhibitors

**DOI:** 10.3389/fphar.2020.00891

**Published:** 2020-06-12

**Authors:** Ying Jin, Zhifei Xu, Hao Yan, Qiaojun He, Xiaochun Yang, Peihua Luo

**Affiliations:** Center for Drug Safety Evaluation and Research of Zhejiang University, College of Pharmaceutical Sciences, Zhejiang University, Hangzhou, China

**Keywords:** targeted therapies, protein kinases, small-molecule kinase inhibitor, cardiotoxicity, incidence

## Abstract

Numerous protein kinases encoded in the genome have become attractive targets for the treatment of different types of cancer. As of January 2020, a total of 52 small-molecule kinase inhibitors (SMKIs) have been approved by the FDA. With the numerous clinical trials and a heavy focus on drug safety, SMKI-induced cardiotoxicity, which is a life-threatening risk, has greatly attracted the attention of researchers. In this review, the SMKIs with cardiotoxicity incidence were described exhaustively. The data were collected from 42 clinical trials, 25 FDA-published documents, seven meta-analysis/systematic reviews, three case reports and more than 50 other types of articles. To date, 73% (38 of 52) of SMKIs have reported treatment-related cardiotoxicity. Among the 38 SMKIs with known cardiotoxicity, the rates of incidence of cardiac adverse events were QT prolongation: 47% (18 of 38), hypertension: 40% (15 of 38), left ventricular dysfunction: 34% (13 of 38), arrhythmia: 34% (13 of 38), heart failure: 26% (10 of 38) and ischemia or myocardial infarction: 29% (11 of 38). In the development process of novel SMKIs, more attention should be paid to balancing the treatment efficacy and the risk of cardiotoxicity. In preclinical drug studies, producing an accurate and reliable cardiotoxicity evaluation model is of key importance. To avoid the clinical potential cardiotoxicity risk and discontinuation of a highly effective drug, patients treated with SMKIs should be proactively monitored on the basis of a global standard. Moreover, the underlying mechanisms of SMKI-induced cardiotoxicity need to be further studied to develop new therapies for SMKI-induced cardiotoxicity.

## Introduction

Protein kinases (PKs) are critical messengers in the regulation of a variety of biological processes. Transferring the γ-phosphate group of adenosine triphosphate (ATP) to substrates affects their substrate protein structure and activity and regulates cell survival, proliferation, apoptosis, migration, transcription, metabolism, and differentiation (Manning et al., 2002; Verkhivker, 2007). Pharmacological and histopathological studies have shown that dysregulation, mutation and overexpression of PKs are closely related to many diseases, including cancer, diabetes mellitus, neurological diseases and cardiovascular diseases (Cohen, 2001). Therefore, PKs appear to be ideal targets for drug development.

Small-molecule kinase inhibitors (SMKIs) are predominantly designed based on the competition for the ATP binding pocket, blocking the catalytic function of PKs (Bogoyevitch and Fairlie, 2007; Backes et al., 2008). This prevents the phosphorylation of downstream substrates and, in turn, protects PK-induced disease progression (Zhang et al., 2009). In addition, more than 5,000 kinds of KIs and SMKIs crystal structures have been determined in recent years, making SMKIs relatively easy for drug development (Wu et al., 2015). Over the past 19 years, the US Food and Drug Administration (FDA) approved up to 52 SMKIs. Tyrosine kinase inhibitors (TKI) represent the majority of all SMKIs, followed by serine/threonine kinase inhibitors and lipokinase inhibitors (Roskoski Jr, 2019). In addition, side effects caused by SMKIs are noteworthy, including gastrointestinal toxicity, hepatotoxicity, cardiovascular toxicity, etc. Here, we focus on the incidence of cardiotoxicity during SMKIs clinical use. On the one hand, the role of PKs in heart and tumor tissue might be dramatically different, and the inhibition of PKs could also abolish normal cardiac function, leading to the occurrence of cardiotoxicity (Chen et al., 2017; Zhang et al., 2017; Miyawaki et al., 2020). On the one hand, the inhibition of kinases could also abolish normal cardiac function due to the dramatically different role of PKs in heart and tumor tissue, which leads to the occurrence of cardiotoxicity (Hasinoff et al., 2017; Livingston et al., 2019). Therefore, clinical research, monitoring and management of SMKI cardiotoxicity are urgently needed.

In contrast to conventional anthracycline chemotherapy, the vast majority of cardiotoxicity induced by SMKIs is reversible. Although SMKI-induced cardiotoxicity has a relatively low incidence and good adaptability, monitoring and controlling cardiotoxicity in a timely and effective manner are essential for these tumors with a favorable prognosis (Cheng and Force, 2010). The cardiotoxicity of SMKIs cannot be generalized, and their toxicity types are not all the same. Common toxicity presentations include the clinical signs and syndromes of cardiac dysfunction and arrhythmia, such as tachycardia, palpitation, QT prolongation and reduction in left ventricular ejection fraction (LVEF) (Lamore et al., 2020). Some SMKIs also manifest direct cardiomyocyte injury, leading to heart failure (HF), myocardial infarction, etc.

In this review, we aimed to outline the cardiotoxicity incidence of FDA-approved SMKIs from the studies evaluated the incidences with respect to cardiac adverse effects of SMKIs. Firstly, we performed a literature search in PubMed-MEDLINE using the following terms: (drug name AND AE type). The names of 52 SMKIs were used in turn as the “drug name”. “cardi* toxicity, adverse effects, QT, left ventricular, heart failure, ischemia, myocardial infarction, arrhythmia, hypertension” were key words used as the “AE type”. The article types comprised clinical trial, meta-analysis, case reports and reviews and the publication dates ranged from January 1998 to January 2020. Then, articles were evaluated according to the following criteria: prospective and retrospective clinical trials of SMKIs; systematic review or meta-analysis evaluating SMKI-related cardiac AEs, case reports informing SMKI-related cardiac AEs and availability of safety data related to cardiotoxicity. Notably, we would investigate the safety data of the drug from FDA-published prescribing information if there were scarce information of cardiotoxicity induced by a certain drug. We concluded the cardiotoxicity incidence and targets of 52 SMKIs approved by the FDA (**Table 1**), and the known incidence of SMKI cardiotoxicity is discussed in detail below.

**Table 1:** Cardiac adverse events of SMKIs with cardiotoxicity.

Initial US FDA approval	Drug	Targets	Cardiac adverse events						
			QT interval prolongation	LV^a^ dysfunction	HF^b^	Arrhythmia	Ischemia or myocardial infarction	Hypertension	Others
2001	Imatinib (Glivec)	Bcr-Abl, PDGFRα, c-KIT	**×**	**○**	**○**	**×**	**×**	**×**	
2005	Sorafenib (Nexavar)	VEGFRs, B-RAF, CSF-1R, PDGFRβ, KIT, FLT3, RET, RET,	**○**	**×**	**×**	**×**	**○**	**○**	
2006	Dasatinib (Sprycel)	Bcr-Abl, PDGFRβ, c-KIT, EPHA2, Src	**○**	**○**	**○**	**○**	**○**	**○**	
2006	Sunitinib (Sutent)	PDGFRα/β, VEGFRs, KIT, FLT3, CSF-1R, RET	**○**	**○**	**○**	**○**	**○**	**○**	Torsade de Pointes
2007	Nilotinib (Tasigna)	Bcr-Abl, PDGFRα, c-KIT, CSF-1R,	**○**	**×**	**×**	**×**	**•**	**×**	
2007	Lapatinib (Tykerb)	EGFR, HER2	**×**	**○**	**○**	**×**	**×**	**×**	
2009	Pazopanib (Votrient)	VEGFRs, PDGFRα/β, FGFR, c-Kit, Itk, Lck, c-Fms	**○**	**•**	**○**	**○**	**○**	**○**	Torsades de pointes
2011	Crizotinib (Xalkori)	ALK, ROS1, MET, RET	**•**	**×**	**×**	**•**	**×**	**×**	
2011	Vemurafenib (Zelboraf)	B-RAF, C-RAF, A-RAF, ACK1	**○**	**•**	**×**	**×**	**×**	**×**	
2011	Vandetanib (Caprelsa)	EGFR, VEGFR, RET, Src	**•**	**×**	**×**	**○**	**×**	**○**	Torsades de pointes
2012	Ponatinib (Iclusig)	Bcr-Abl, VEGFR, PDGFR, FGFR, Src, KIT, RET, FLT3	**×**	**×**	**○**	**○**	**×**	**○**	
2012	Axitinib (Inlyta)	VEGFRs	**×**	**×**	**○**	**×**	**×**	**•**	
2012	Cabozantinib (Cabometyx)	B-RAF, MET, VEGFRs, AXL, RET, ROS1, c-KIT, TrkB, FLT-3	**×**	**×**	**×**	**×**	**×**	**•**	
2012	Regorafenib (Stivarga)	VEGFRs, B-RAF, KIT, RET, PDGFRα/β, FGFR1/2, Raf-1, CSF1R, DDR2, TrkA	**×**	**×**	**×**	**×**	**○**	**○**	
2013	Dabrafenib (Tafinlar)	B-RAF C-RAF, SIK1	**×**	**○**	**×**	**×**	**×**	**×**	peripheral oedema
2013	Trametinib (Mekinist)	B-RAF, MEK1/2	**×**	**○**	**×**	**×**	**×**	**×**	peripheral oedema
2013	Ibrutinib (Imbruvica)	BTK	**×**	**×**	**×**	**○**	**×**	**○**	
2014	Ceritinib (Zykadia)	ALK, IGF-1R, InsR, ROS1	**○**	**×**	**×**	**○**	**○**	**×**	
2014	Nintedanib (Ofev)	PDGFR, FGFRs, VEGFRs, et.al	**×**	**×**	**×**	**×**	**○**	**×**	
2015	Osimertinib (Tagrisso)	EGFR, HER2/3/4, ACK1, Blk	**•**	**○**	**○**	**○**	**○**	**×**	pericardial effusion
2015	Alectinib (Alecensa)	ALK, LTK, GAK, RET	**○**	**×**	**×**	**○**	**×**	**×**	
2015	Cobimetinib (Cotellic)	B-RAF, MEK1/2	**○**	**•**	**×**	**×**	**×**	**×**	
2015	Lenvatinib (Lenvima)	VEGFRs, FGFRs, PDGFRα, KIT, RET	**•**	**○**	**○**	**×**	**×**	**○**	
2017	Brigatinib (Alunbrig)	ALK, ROS1, IGF-1R, FLT-3, EGFR	**×**	**×**	**×**	**○**	**×**	**○**	
2017	Ribociclib (Kisqali)	CDK4/6	**○**	**×**	**×**	**×**	**×**	**×**	
2017	Midostaurin (Rydapt)	FLT3, KIT, PDGFRα/β, VEGFR2, PKC	**×**	**×**	**×**	**×**	**×**	**○**	pericardial effusion
2017	Abemaciclib (Verzenio)	CDK4/6	**×**	**×**	**×**	**×**	**×**	**×**	cardiovascular malformations and variations
2017	Acalabrutinib (Calquence)	BTK	**×**	**×**	**×**	**○**	**×**	**×**	
2017	Copanlisib (Aliqopa)	PI3K-α/δ	**×**	**×**	**×**	**×**	**×**	**•**	
2018	Lorlatinib (Lorbrena)	ALK, LTK, ROS1	**×**	**×**	**×**	**×**	**○**	**×**	PR interval prolongation atrioventricular block
2018	Encorafenib (Braftovi)	B-RAF	**○**	**○**	**×**	**×**	**×**	**×**	
2018	Binimetinib (Mektovi)	MEK1/2	**○**	**○**	**×**	**×**	**×**	**×**	
2018	Gilteritinib (Xospata)	FLT3, AXL	**○**	**×**	**×**	**×**	**×**	**×**	
2018	Fostamatinib (Tavalisse)	SYK	**×**	**×**	**×**	**×**	**×**	**○**	
2019	Zanubrutinib (Brukinsa)	BTK	**×**	**×**	**×**	**○**	**×**	**×**	
2019	Fedratinib (Inrebic)	JAK2	**×**	**×**	**○**	**×**	**×**	**×**	cardiogenic shock
2019	Entrectinib (Rozlytrek)	ALK, ROS1, TrkA/B/C	**○**	**×**	**×**	**×**	**×**	**×**	
2019	Erdafitinib (Balversa)	FGFR	**×**	**×**	**×**	**×**	**○**	**×**	

a LV, left ventricular.

b HF, heart failure.

**○**The cardiac adverse event has been reported.

**•**The reported incidence of the cardiac adverse event is ≥10%.

**×**The cardiac adverse event has not been reported.

Abbrs in the table:

ACK1, activated cdc42-associated kinase 1; ALK, anaplastic lymphoma kinase; AXL, a tyrosine-protein kinase receptor from myeloid leukemia cells; BCR-ABL: breakpoint cluster region-Abelson fusion protein; BTK, Bruton's tyrosine kinase; CDK, cyclin-dependent kinase; c-Fms, transmembrane glycoprotein receptor tyrosine kinase; c-KIT, cellular membrane tyrosine kinase receptor; CSF1R, colony stimulating factor 1 receptor; DDR, discoidin domain receptors; EGFR, epidermal growth factor receptor; EPHA, ephrin type-A receptor; FGFR, ﬁbroblast growth factor receptor; FLT3, fms related tyrosine kinase 3; GAK, cyclin-g associated kinase; HER, human epidermal growth factor receptor; IGF-1R, insulin-like growth factor-1 receptor; InsR, insulin receptor; Itk, interleukin-2 receptor-inducible T-cell kinase; JAK, janus kinase; Lck, lymphocyte-specific protein tyrosine kinase; LTK, leukocyte receptor tyrosine kinase; MEK, mitrogen-activated protein extra-cellular regulated-kinase; MET, or HFG, hepatocyte growth factor; PDGFR, platelet-derived growth factor receptor; PI3K, phosphatidylinositol-3-kinase; PKC, protein kinase C; RAF, Raf kinase; RET, rearranged during transfection tyrosine kinase; ROS1, c-ros oncogene 1 receptor kinase; SIK, salt-inducible kinase; Src, proto-oncogene tyrosine-protein kinase; SYK, spleen tyrosine kinase; Trk, tropomyosin-related kinase; VEGFR, vascular endothelial growth factor receptor.

## Chronic Myeloid Leukemia Associated SMKIs: Imatinib, Nilotinib, Dasatinib and Ponatinib

Chronic myeloid leukemia (CML) is a common hematological malignancy driven by the oncogene BCR-ABL1 (Quintás-Cardama and Cortes, 2009). To date, three generations of SMKIs have been developed. Imatinib was the first SMKI approved for the treatment of CML, which could significantly improve the 5-year survival rates of patients (Druker et al., 2006). Second-generation SMKIs, nilotinib and dasatinib, were indicated for the treatment of CML after imatinib failure from the outset (Branford et al., 2009). Some later studies showed that second-generation SMKIs have more potent kinase inhibitory activity than imatinib; hence, they were approved as the first-line therapy (Shah et al., 2004; Larson et al., 2012; Jabbour et al., 2014). Ponatinib was the third-generation SMKI and the only kinase inhibitor targeted to the BCR-ABL1^T315I^ mutation (Cortes et al., 2012; Cortes et al., 2013).

### Imatinib

In 2003, the International Randomized Study of Interferon versus STI571 (IRIS) trial (O'Brien et al., 2003) was carried out in a double-blind and randomized manner. Researchers compared imatinib and interferon alpha plus cytarabine in CML, and the study finally determined that the incidence of HF was approximately 1% in imatinib 400 mg group. Imatinib-induced cardiotoxicity has gradually gained more attention. A retrospective analysis was conducted by Atallah et al. (2007) to investigate the cardiotoxicity of 1,276 patients treated with imatinib at dose of 400 to 1000 mg for hematologic malignancies in clinical trials. They summarized the eight-year clinical outcomes at the M. D. Anderson Cancer Centre; less than 0.1% of patients showed left ventricular (LV) dysfunction, and 1.7% of patients showed symptoms correlating with HF. In the same year, another retrospective analysis based on a trail of 946 patients at a daily dose of imatinib 400 mg or 800 mg was published (Verweij et al., 2007). By combining patient history with physical examination, the data showed that the incidence of HF was approximately 1%.

### Dasatinib

As reported in clinical trials, dasatinib-related cardiac toxicities include QT interval prolongation, LV dysfunction, arrhythmia, HF, arterial ischemic and pulmonary hypertension (PH) (Ilene Galinsky and Susan Buchanan MS, 2009). With a five-year follow-up, Dasatinib Versus Imatinib Study in Treatment-Naive CML Patients (DASISION) study proposed that the risk of arterial ischemic events was 5% during dasatinib treatment at a daily dose of 100 mg (Kantarjian et al., 2012). Bristol-Myers Squibb, the manufacturer of dasatinib, reported that continuous administration over 6 months with dasatinib could cause a 4% incidence of HF or LV dysfunction (Garcia-Alvarez et al., 2010). A randomized phase 2 trial reported that the rates of superficial edema and fluid retention in patients receiving dasatinib 100 mg/day were up to 15 and 30%, respectively, which might be the symptoms of heart injury (Kantarjian et al., 2007). Dasatinib could potentially prolong QT interval of patients according to a reviewed clinical profile of dasatinib-treated patients with imatinib-resistance (Wong, 2009). The incidence of dasatinib-induced QT prolongation was less than 1%, but still should be monitored during therapy because of severe effects caused by QT prolongation. Spechbach et al. (2013) reported a case in which a 54-year-old patient on second-line treatment with dasatinib 100 mg daily showed a cardiac adverse event (AE) of ventricular arrhythmias. In addition, as described in a study that focused on incident cases of dasatinib-associated PH in France, dasatinib, dose of which ranged from 70 to 140 mg daily, may induce severe precapillary PH and the incidence of PH occurring in patients treated with dasatinib was approximately 0.45% (Montani et al., 2012).

### Nilotinib

In a phase 1 clinical trial, nilotinib was reported to induce a prolongation ranging from 5 to 15 ms in the QT interval (Kantarjian et al., 2006). In the study, doses of nilotinib ranged from 50 to 1,200 mg once daily or 400 to 600 mg twice daily. Torsade de pointes, which is life-threatening and able to cause syncope, might be induced by nilotinib-related QT interval prolongation. Hence, avoidance of medicinal products with known QT interval prolongation potential and careful observation of detailed electrocardiograms during nilotinib therapy were necessary. Ischemic heart disease-related cardiac events were another symptom shown in a clinical trial (Larson et al., 2012). After an average time on nilotinib therapy of 60 months, the incidences of ischemic heart disease-related cardiac events in the nilotinib 300 mg arm and 400 mg arm were 9.3 and 15.2%, respectively (Larson et al., 2012).

### Ponatinib

Among several effective SMKIs for CML treatment, ponatinib has the highest risk of cardiotoxicity, including congestive heart failure (CHF), cardiac arrhythmias and hypertension (Dorer et al., 2016). According to the prescribing information of ponatinib^1^, in clinical trials, the incidence of all-grade CHF or LV dysfunction in ponatinib-treated patients at a recommended dose of 45 mg/day was 7%; and serious CHF or LV dysfunction occurred in 4% of ponatinib-treated patients with four fatalities (U.S. Food and Drug Administration, 2012b). Besides, treatment-related symptomatic hypertension, including hypertensive crisis, was reported as a serious adverse reaction in 2% of patients. Clinical trials designed to compare the treatment effect in CML between ponatinib and imatinib failed for serious ponatinib cardiac toxicity (Moslehi and Deininger, 2015). In the phase 2 ponatinib CML evaluation trial, ponatinib was shown to have dose-dependent cardiotoxicity in 267 evaluated patients at a starting dose of 45 mg once daily (Cortes et al., 2018). Among the ponatinib-treated CML patients participating in clinical trials, 31% reported arterial occlusive events in the 5-year follow-up. Additionally, 4% of patients reported cardiac AE of atrial fibrillation (AF), and 3% of which were showed to angina pectoris.

## Non-Small Cell Lung Cancer Associated SMKIs: Crizotinib, Alectinib, Osimertinib, Ceritinib, Lorlatinib and Brigatinib

The kinase targets in non-small cell lung cancer (NSCLC) include epidermal growth factor receptor (EGFR), anaplastic lymphoma kinase (ALK) and ROS proto-oncogene 1 (ROS1) (Korpanty et al., 2014). Crizotinib, alectinib, ceritinib, lorlatinib and brigatinib are inhibitors of ALK that were developed principally for the treatment of NSCLC. These five TKIs were indicated for the treatment of patients with ALK-positive NSCLC. Apart from that, crizotinib, ceritinib and lorlatinib were also effective for the treatment of patients with ROS1, which retains constitutive kinase activity in NSCLC. Osimertinib, which targets EGFR exon 19 deletions, exon 21 L858R mutations and T790M mutations, was approved as a third-generation TKI indicated for the first-line treatment of patients with metastatic NSCLC whose tumors were resistant to the first- and second- generation EGFR-TKIs (Wu and Shih, 2018).

### Crizotinib

Two main cardiac AEs: QT interval prolongation and bradycardia occurred in crizotinib-treated patients in clinical practice (Tartarone et al., 2015). The safety results of crizotinib in a large-scale multinational trial (PROFILE 1005), which enrolled 1,066 patients receiving crizotinib 250 mg twice daily, reported that corrected QT (QTc) prolongation as a cardiac AE occurred in 4% of crizotinib-treated patients, and 12 patients reached grade 3 severity. In 5% of patients, a maximum Fridericia's correction QT (QTcF) change from baseline of greater than 60 ms was observed. According to the authors, the frequency of crizotinib induced QTc prolongation is similar to that in previous reports (Shaw et al., 2013; Lamore et al., 2020). Additionally, bradycardia was reported in several clinical trials with crizotinib-treated patients at a starting dose of 250 mg twice daily. as a treatment-related AE. Notable, 13% of crizotinib-treated patients experienced bradycardia of a pulse rate <50 beats/min, most of which were grade 1 or 2 in this study (Blackhall et al., 2017). Bradycardia was both reported in the phase 3 study (PROFILE 1014) in ALK-positive NSCLC (Solomon et al., 2016) and the phase 1 study (PROFILE 1001) in ROS1-rearranged advanced NSCLC (Shaw et al., 2019). The PROFILE 1014 trial and the PROFILE 1001 trial reported a relatively high incidence of bradycardia: 14% of patients and 21% of patients, respectively. Of note, there was significantly different incidences between the crizotinib (14%) arm and the chemotherapy arm (1%) in the PROFILE 1014 study (Blackhall et al., 2017).

### Ceritinib

The cardiotoxicity of ceritinib was similar but not identical to that of crizotinib. The Study X2101 preliminary analysed the safety of ceritinib, which included 246 patients with NSCLC and nine patients with other cancers who were treated with ceritinib 750 mg daily. Treatment-related AEs included prolonged QT interval in 4% patients, bradycardia in 3% patients, and fatal cardiac tamponade in one patient (Khozin et al., 2015). The open-label, phase 1 trial performed in patients with ALK-rearranged NSCLC reported that ceritinib treated-related QTc interval prolongation of changing greater than 60 ms from baseline occurred in 3% of patients at a recommended dose of 750 mg/day (Kim et al., 2016). Shaw et al. (2014) observed one case of asymptomatic grade 3 prolongation of the QTc interval that was possibly related to ceritinib at a dose of 700 mg once daily.

### Osimertinib

Osimertinib was reported to be more severe cardiotoxic than EGFR-TKIs. According to a retrospective review of osimertinib-induced cardiotoxicity, osimertinib-induced cardiotoxicity includes QTc interval prolongation, LVEF decline, cardiac failure, arrhythmia, myocardial infarction and pericardial effusion (Anand et al., 2019). A double-blind, phase 3 trial (Soria et al., 2018) enrolled 279 patients at a dose of osimertinib 80 mg once daily reported that 10% of osimertinib-treated patients experienced changes of the QT interval. A maximum change of 17.7 ms from baseline in the median QTcF was reported at week 12 in the osimertinib group. In a phase 2 study (Goss et al., 2016) included 472 patients with EGFR T790M-positive mutations, osimertinib-related QT prolongation was recorded in 5% of patients at all-grade, and it was one of the most common severe AEs occurred in 2% of patients at grades 3–4. The grades 3–4 QT prolongation eventually led to a dose interruption or reduction. Furthermore, a randomized phase 3 study compared osimertinib versus chemotherapy for EGFR T790M positive advanced NSCLC and determined that LVEF occurred in 5% of osimertinib-treated patients at a dose of 80 mg/day, with a median time of 5.5 months to outset of decreased LVEF (Mok et al., 2017). In addition to QTc interval prolongation and LVEF decline, the incidences for cardiac failure, AF, myocardial infarction, and pericardial effusion were about 5.4, 4.0, 1.6, and 8.2%, respectively (Mok et al., 2017). Compared with other TKIs, the incidences for QT prolongation, AF and cardiac failure induced by osimertinib were higher (Mok et al., 2017).

### Alectinib

QT interval prolongation and bradycardia were descripted as two main cardiac AEs in the prescribing information of alectinib from the FDA (U.S. Food and Drug Administration, 2015a). As analyzed in pivotal phase 2 trials (NP28673 and NP28761) (Morcos et al., 2017), with patients receiving alectinib 600 mg twice daily, 6% of patients experienced prolongation of QT interval or arrhythmia potentially associated with alectinib treatment; 4% of patients experienced sinus bradycardia, which was possibly associated with QT interval prolongation or arrhythmia. 8% of patients experienced other cardiac function disorder related AEs, most of which were grades 1–2, included bradycardia, palpitations, myocardial infarction, and pericardial effusion. Toyoaki Hida et al. (Hida et al., 2017) performed a phase 3 trial to directly compare the efficacy and safety of alectinib with crizotinib. Alectinib 300 mg twice daily or crizotinib 250 mg twice daily were assigned equally and randomly to patients. In the alectinib arm, all-grade QTc prolongation occurred in 3% of patients, grades 3–4 QTc prolongation occurred in 2% of patients; and sinus bradycardia occurred in 1% of patients. In the crizotinib arm, the incidences of all-grade and grades 3–4 QTc prolongation were 14 and 7%, respectively; and sinus bradycardia occurred in 6% of patients. Alectinib seems to have a better cardiac safety profile than that of crizotinib.

### Lorlatinib

The prescribing information of the FDA for lorlatinib described that drug-induced PR interval prolongation and atrioventricular (AV) block was reported in patients receiving lorlatinib (U.S. Food and Drug Administration, 2018a). As described, 1% of patients experienced AV block and 0.3% of patients experienced grade 3 AV block that led to pacemaker placement. In study B7461001, of 295 patients with ALK-positive metastatic NSCLC who received lorlatinib 100 mg orally once daily, 0.7% occurred myocardial infarction which led to permanently discontinued lorlatinib treatment (Fala, 2019).

### Brigatinib

In the ALTA trial, bradycardia and hypertension were reported as cardiac AEs induced by brigatinib (Markham, 2017). As described in the prescribing information of the FDA for brigatinib, 5.7 and 7.6% of patients experienced heart rates less than 50 beats/min in the 90 mg arm and the 90–180 mg arm, respectively; and one patient (0.9%) was experienced grade 2 bradycardia in the 90 mg arm (U.S. Food and Drug Administration, 2017b). A randomized phase 3 trial, which compared brigatinib versus crizotinib in the treatment for patients with ALK-positive NSCLC, reported that incidence of hypertension was about 23% in the brigatinib 180 mg arm and 7% in the crizotinib 250 mg arm (Camidge et al., 2018).

## Melanoma Associated SMKIs: Dabrafenib Plus Trametinib, Vemurafenib Plus Cobimetinib, and Encorafenib Plus Binimetinib

In the past few decades, melanoma incidence has dramatically increased throughout the world, and it is the most malignant skin cancer worldwide (Korpanty et al., 2014). In addition to surgical resection, targeted therapy and immunotherapy have been particularly available in the treatment of melanoma with a high rate of recurrence and metastasis (Balch et al., 2010; Ugurel et al., 2016). The combination of BRAF and MEK inhibitors is the most common melanoma targeted therapy. As described below, we collected the cardiac toxicity of three approved combinations of SMKIs.

### Dabrafenib Plus Trametinib

The combination of dabrafenib and trametinib has been extensively studied, and several clinical trials that combined dabrafenib 150 mg twice daily and trametinib 2 mg once daily have been performed. In 2014, a phase 3 trial was carried out that involved 947 patients with unresectable advanced melanoma with a mutation for BRAF V600, V600E or V600K. In this work, Long et al. (2014) reported that peripheral oedema occurred in 11% of patients and decreased ejection fraction (EF) in 4% of patients. Updated results published in 2015 showed that all-grade of decreased EF were happened in 4% patients, grade 2 was 3% and grade 3 was 1% (Long et al., 2015). The population of sample sizes would make the results inconsistent with other trials. The work published by Planchard et al. (2016) registered that the incidences of EF decrease were 3% in grades 1–2 and 6% in grade 3, and cardiac arrest grade 5 was 3%. Randomized trials of combination therapy in five-year data could reflect the real state of patients with durable administration. Researchers evaluated 559 patients and reported that 4% of patients had to discontinue the therapy due to the decreased EF (Robert et al., 2019).

### Vemurafenib Plus Cobimetinib

The clinical trials of vemurafenib plus cobimetinib versus vemurafenib plus placebo in metastatic melanoma (NCT01689519) were conducted in 2014. The 495 patients were randomly divided into combination therapy and vemurafenib plus placebo groups. The data registered were as follows: nine patients had all-grade of QT interval prolongation, and among them, one patient (< 1%) reported grade 3 QT interval prolongation (Enrico et al., 2018). Similar research was updated in another phase 3 clinical trial by Ascierto et al. (2016). Multiple types of cardiac disorders were presented in patients treated with combination therapy. QT interval prolongation in all-grade occurred in 5% of patients, and 3 patients showed grade 3 QT interval prolongation. Decreased EF occurred in 12% of patients receiving vemurafenib 960 mg twice daily plus cobimetinib 60 mg once daily. As reported, one patient died from cardiac arrest, which might be the result of cardiotoxicity (Ascierto et al., 2016).

### Encorafenib Plus Binimetinib

Another combination therapy treatment was encorafenib and binimetinib, which was studied in the COLUMBUS (study of encorafenib plus binimetinib versus encorafenib versus vemurafenib in metastatic melanoma) trial (Dummer et al., 2018), which screened patients with advanced unresectable or metastatic melanoma which is BRAF V600 mutation positive. According to the profile of 192 patients receiving encorafenib 450 mg once daily plus binimetinib 45 mg twice daily, researchers found that the incidence of all-grade of LV dysfunction was 6%, and these patients had a dose reduction. Another study published in 2019 updated the outcomes of the COLUMBUS with minor differences. The data showed that LV dysfunction was reported in 8% of patients, and grades 1–2 and grade 3 LV dysfunction were 6% and 2%, respectively (Gogas et al., 2019).

## Breast Cancer Associated SMKIs: Lapatinib, Ribociclib and Abemaciclib

Breast cancer targeted therapy drugs block the action of abnormal kinases, such as human epidermal growth factor receptor 2 (HER2) and cyclin-dependent kinase (CDK) 4 and 6, which stimulate the growth of breast cancer cells in specific ways (Gu et al., 2016). Lapatinib targets HER2 and it was approved as a first-line therapy for patients who advanced or metastatic breast cancer with overexpressed HER2. Ribociclib and abemaciclib is an SMKI that specifically inhibits CDK4/6, which overexpresses in hormone receptor (HR)-positive breast cancer. The FDA approved ribociclib and abemaciclib a first-line therapies for the treatment of postmenopausal women whose tumors are HR-positive, HER2-negative.

### Lapatinib

Perez et al. (2008) analyzed the cardiac safety of lapatinib based on the prospective data of 44 clinical studies that enrolled 3,689 patients treated with lapatinib. According to the study, patients treated with lapatinib showed low risk of cardiotoxicity, and cardiac AEs were usually asymptomatic and reversible, for example, LVEF decline. These data also reported a very low rate of symptomatic CHF (0.2%) (Perez et al., 2008). Moreover, a randomized study of lapatinib monotherapy versus combination therapy also reported that the cardiotoxicity of lapatinib was low. The incidences of symptomatic and asymptomatic cardiac AEs were 0.7 and 1.4%, respectively, in the lapatinib 1,000 mg group; and no treatment-related cardiac deaths occurred among patients (Blackwell et al., 2010).

### Ribociclib

The prescribing information from the FDA for ribociclib stated that the drug potentially increased the cardiotoxicity risk of QT interval prolongation in a concentration-dependent manner (U.S. Food and Drug Administration, 2017d). A total of 1,054 patients were included in three clinical trials: MONALEESA-2 (Hortobagyi, 2018), MONALEESA-3 (Slamon et al., 2018), and MONALEESA-7 (Tripathy et al., 2018). As summarized, 6% of patients who were treated with the combination of ribociclib 600 mg plus an aromatase inhibitor or fulvestrant reported a greater than 60 ms prolongation of QTc interval, and 1% of patients reported a greater than 500 ms prolongation of QTc interval. FDA approval of ribociclib was based on a clinical trial, MONALEESA-2, which reported a 3.3% incidence of QTc interval prolongation in patients receiving ribociclib 600-mg daily, and one ≥65 year-old patient experienced grade 3 QTc interval prolongation (Hortobagyi, 2018).

### Abemaciclib

Scarce information of abemaciclib-related cardiotoxicity was reported. As described in the prescribing information, cardiovascular malformations and variations occurred in a preclinical animal trial, which warned of the risks of cardiotoxicity in patients treated with abemaciclib (U.S. Food and Drug Administration, 2017f). Moreover, in Studies 0020 and 0021, 30% of patients receiving abemaciclib 250 mg once a month with anastrozole 1 mg once daily experienced AEs of cardiovascular system.

## Renal Cell Carcinoma Associated SMKIs: Sorafenib, Sunitinib, Pazopanib, Axitinib, Lenvatinib, and Cabozantinib

The SMKIs in this group are multitarget kinase inhibitors. The common targets of these SMKIs include vascular endothelial growth factor receptors 1, 2 and 3 (VEGFRs) and platelet-derived growth factor receptor (PDGFR), which are both present in renal cell carcinoma (RCC) (Porta and Schmidinger, 2019). Sorafenib is the approved drug for the treatment of patients with RCC, unresectable hepatocellular carcinoma (HCC) and thyroid carcinoma (DTC). Sunitinib and pazopanib are first-line therapies for metastatic RCC. In addition to metastatic RCC, sunitinib also targets colony stimulating factor receptor (CSFR) which was indicated for a treatment target of gastrointestinal stromal tumor (GIST) and pancreatic neuroendocrine tumor. Pazopanib is also an inhibitor of fibroblast growth factor receptor (FGFR) and c-Fms. Apart from metastatic RCC, pazopanib was indicated for the treatment of patients with advanced soft tissue sarcoma. Axitinib is a relatively novel inhibitor, which was indicated for the treatment of advanced RCC that still progressed after at least one prior systemic therapy, including therapy with the drugs above. Cabozantinib targets hepatocyte growth factor (MET) and tyrosine-PKs receptor from myeloid leukemia cells (AXL) in addition to VEGFRs, and it was indicated for the treatment of patients with HCC after sorafenib failure. Lenvatinib targets FGFR and was approved for the treatment of patients with RCC in combination with everolimus, DTC and HCC.

### Sorafenib

The prescribing information of the FDA for sorafenib described that the drug could increase the risk of cardiac ischemia or infarction and QT interval prolongation (U.S. Food and Drug Administration, 2005). A phase 1 open-label study evaluated the cardiotoxicity of sorafenib. In this study, 6.5% of patients receiving sorafenib 400 mg twice daily experienced a decreased LVEF; and 31 patients showed a less than 60 ms change from baseline in QTc, indicating that the QTc interval is prolonged moderately (Tolcher et al., 2011). In a sorafenib phase 3 trial, patients were randomly treated with sorafenib 400 mg twice daily or placebo in a 1:1 ratio. The incidence of cardiac ischemia or infarction in the sorafenib group (3%) was significantly higher than that in the placebo group (< 1%). Notably, cardiac ischemia or infarction was reported as one of the serious AEs leading to hospitalization or death in at least 2% of patients (Escudier et al., 2007). According to a long-term safety analysis of a phase 3 sorafenib clinical trial, higher incidence of cardiac AEs was reported in the sorafenib group than the placebo group. The rate of treatment-related cardiac ischemia or infarction was 2%, and LV systolic dysfunction was 1% (Hutson et al., 2010). Hypertension, which potentially leads to severe cardiovascular events, is one of the common AEs of sorafenib. As reported in a systematic review that analyzed a total of 4599 patients treated with sorafenib, the overall incidences of all-grade and grades 3–4 hypertension were 23.4 and 5.7%, respectively (Wu et al., 2008).

### Sunitinib

The prescribing information of the FDA for sunitinib described treatment-related cardiac AEs, including prolonged QT interval, torsade de pointes, LVEF declines, myocardial ischemia or infarction and cardiac failure (U.S. Food and Drug Administration, 2006). Michael S. Ewer et al. (Ewer et al., 2014) defined cardiac risk in patients receiving sunitinib 50 mg once daily versus interferon-alpha/placebo through a retrospective adjudication of comprehensive cardiac AEs from two phase 3 trials. The study indicated that sunitinib-related cardiovascular incidence rates of symptomatic LVEF and asymptomatic LVEF were 0.4 and 1.1%, respectively; and rates of both symptomatic and asymptomatic LVEF declines observed in patients in the sunitinib group were significantly higher than those in the interferon-alfa/placebo group. About 1.4% of individual sunitinib-treated patients experienced a cardiac event in symptoms possibly related to HF. Symptomatic LVEF decrease-related sudden death were adjudicated in one patient (Ewer et al., 2014). The LV dysfunction might be exacerbated or partially caused by hypertension. Zhu et al. (2009) systematically reviewed that the incidences of all-grade and high-grade hypertensions among 4999 sunitinib-treated patients were 21.6 and 6.8%, respectively. Richards et al. (2011) defined the overall incidence of sunitinib-induced CHF. Based on data from 6,935 enrolled patients, the study reported that the overall incidences of all- and high-grade CHF in sunitinib-treated patients were 4.1 and 1.5%, respectively. Usually, patients in these studies take the starting dose of sunitinib 50 mg once daily.

### Pazopanib

The prescribing information of the FDA for sunitinib described that pazopanib had risk of inducing prolonged QT interval and torsades de pointes, cardiac dysfunction such as CHF and decreased LVEF (U.S. Food and Drug Administration, 2009). As summarized in the information, in the RCC trials of pazopanib at a dose of pazopanib 800 mg once daily, the incidences of drug-related conduction disturbances included QT prolongation and torsades de pointes were 2% and less than 1%, respectively. According to the results of phase 3 trials, pazopanib-related arterial thrombotic events include myocardial infarction or ischemia, cerebrovascular accident, and transient ischemic attack at incidences of 2, < 1 and <1%, respectively (Sternberg et al., 2010). Pinkhas et al. (2017) assessed the cardiotoxicity incidence with pazopanib at a starting dose of 800 mg/d in a clinical setting, and discovered that nearly 70% of patients in the study developed cardiotoxicity. Hypertension, cardiomyopathy and cardiac dysrhythmias occurred in patients treated with pazopanib. The study reported that QTc interval prolongation was the most common AE and represented 23% of all cardiac AEs. The pazopanib treatment-related QTc interval prolongation had a median increase of 16 ms. Five of seven patients who had LVEF assessments during pazopanib treatment occurred an absolute decline in LVEF, and two of these five patients developed clinically significant declines (greater than 10%) in LVEF. The patient who experienced the highest degree of LVEF decline developed significant cardiac comorbidities, including LV dysfunction. Two patients developed acute HF within 30 days of initiating pazopanib treatment, and one of them progressed to fatal cardiogenic shock (Pinkhas et al., 2017).

### Axitinib

As reported in the prescribing information of the FDA, hypertension, including hypertensive crisis, and cardiac failure have been observed (U.S. Food and Drug Administration, 2012c). According to the information, in a clinical study, 359 patients with RCC at a starting dose of 5 mg axitinib twice daily were included. All-grade and grades 3–4 cardiac failure occurred in 2 and 1% of patients, respectively. Moreover, it should be noted that poorly controlled hypertension is capable of inducing serious cardiovascular events. The incidence of hypertension was analyzed in a randomized phase 3 trial enrolled patients with advanced RCC receiving axitinib 5 mg twice daily. The outcomes indicated that 42% of patients developed all-grade hypertension, and 11% of patients developed grade ≥3 hypertension (Motzer et al., 2013).

### Cabozantinib

Cardiac toxicity of cabozantinib has rarely been reported. However, potential cardiac AEs might be induced by hypertension and thrombotic events. According to the prescribing information, hypertension occurred in 36% of patients; and thromboembolism was observed in 7% of patients receiving cabozantinib 60 mg once daily (U.S. Food and Drug Administration, 2012a), suggesting the possibility of developing an acute myocardial infarction.

### Lenvatinib

Two main cardiac AEs were observed: QT interval prolongation and cardiac dysfunction. As described in the prescribing information, study SELECT (lenvatinib-treated patients with DTC at a dose of 24 mg once daily), Study 205 (lenvatinib-treated patients with RCC at a dose of 18 mg once daily) and Study REFLECT (lenvatinib-treated patients with HCC at a dose of 8 mg or 12 mg once daily) stated that QTc interval prolongation occurred in 9, 11 and 8% of patients, respectively; and QTc interval prolongation of greater than 500 ms was observed in 2, 6 and 2% of patients, respectively (U.S. Food and Drug Administration, 2015b). According to an update on cardiovascular safety of tyrosine kinase inhibitors including lenvatinib, 7% of lenvatinib-treated patient experienced cardiac dysfunction and 2% is grade 3 or higher. Most of these patients were diagnosed by finding of EF decline that occurred in 2% of lenvatinib-treated patients compared with none in patients who received placebo (Shah and Morganroth, 2015). Furthermore, hypertension, which could potentially induce cardiac AEs, occurred in 73% of lenvatinib-treated patients compared with 15% of placebo-treated patients in the phase 3 study of lenvatinib in study SELECT (Wirth et al., 2018).

## Other Kinds of SMKIs: Vandetanib, Regorafenib, Ibrutinib, Nintedanib

### Vandetanib

Vandetanib targets both EGFR and VEGFR tyrosine kinases and was indicated for the treatment of symptomatic or progressive medullary thyroid cancer (MTC) in patients. As stated in the prescribing information of vandetanib, QT interval prolongation is one of the major cardiac AEs; torsades de pointes and sudden death have been reported in vandetanib-treated patients (U.S. Food and Drug Administration, 2011). In a randomized, double-blind phase 3 trial, patients with advanced MTC were randomly assigned in a 2:1 ratio to receive vandetanib 300 mg daily or placebo. The trial reported that QTc prolongation occurred in 8% of patients, and these was a single instance of arrhythmia and acute cardiac failure in one patient (Wells et al., 2010). As analyzed in a review that nine trials included 2,188 vandetanib-treated patients, 18.0 and 12.0% of patients with MTC experienced all-grade and high-grade QTc interval prolongation, respectively (Zang et al., 2012). Furthermore, Qi et al. performed a meta-analysis of 11 clinical trials enrolled 3154 vandetanib-treated patients, and summarized incidences of all-grade and high-grade hypertension in patients were 24.2 and 6.4%, respectively (Qi et al., 2013).

### Regorafenib

Regorafenib is a multikinase inhibitor of RET, VEGFRs, KIT, PDGFR-α/β, FGFR1, FGFR2, TIE2, DDR2, TrkA, Eph2A, RAF-1, BRAF, SAPK2, PTK5, Abl and CSF1R. Regorafenib was approved for the treatment of patients with colorectal cancer, GIST and HCC. The prescribing information for regorafenib indicated that there is a risk of cardiac ischemia and infarction in patients treated with the drug (U.S. Food and Drug Administration, 2012d). In randomized placebo-controlled trials, the incidence of myocardial ischemia and infarction was 0.9% in the regorafenib group compared with 0.2% in the placebo group. As reported in a single-group phase 2 trial, grade 4 treatment-related cardiac ischemia or infarction occurred in 4% of patients receiving regorafenib 160 mg once daily (Eisen et al., 2012). Besides, Hsiao et al. (2016) presented the case of a 72-year-old man with a metastatic GIST who received regorafenib therapy and who experienced recurrent episodes of myocardial injury during AF. In addition to cardiac ischemia and infarction, hypertension is one of the major AEs of regorafenib. According to the description of a systematic review, poorly controlled regorafenib-induced hypertension may lead to serious cardiac AEs and dose reduction or discontinuity; the overall incidences of all-grade and high-grade hypertension were about 44.4 and 12.5%, respectively (Wang et al., 2014).

### Ibrutinib

Ibrutinib is a covalent inhibitor of Bruton's tyrosine kinase (BTK) and it is indicated for the treatment of patients with mantle cell lymphoma (MCL), chronic lymphocytic leukemia, small lymphocytic lymphoma, Waldenström's macroglobulinemia and marginal zone lymphoma. According to the prescribing information for ibrutinib, 6 to 9% of patients treated with ibrutinib experienced AF and atrial flutter (U.S. Food and Drug Administration, 2013). Patients with pre-existing hypertension, acute infections, and AF are at a higher risk of experiencing ibrutinib-induced toxicity. Darryl P. Leong (Leong et al., 2016) performed a meta-analysis that evaluated the risk of AF with ibrutinib use in a systematic review, reporting the relative risk of ibrutinib-related AF was 3.9% among four randomized trials of ibrutinib. Compared with the comparator, there is an increased risk of AF in ibrutinib-treated patients (Leong et al., 2016). In addition, as reported in ibrutinib randomized controlled clinical trials, Brown et al. (2017) found that approximately 6.5% of ibrutinib-treated patients at a dose of 420 mg once daily developed AF, compared with 1.6% of comparator-treated patients.

### Nintedanib

Nintedanib is a multiple-target tyrosine kinase inhibitor that targets PDGFR, VEGFR, FGFR, and Src family kinase. The drug is indicated for the treatment of idiopathic pulmonary fibrosis. The prescribing information for nintedanib described that arterial thromboembolic events, which could potentially induce myocardial infarctions, occurred in 2.5% of nintedanib-treated patients at a dose of 150 mg twice daily and 0.8% of placebo-treated patients, respectively (U.S. Food and Drug Administration, 2014). Luca Richeldi et al. evaluated the efficacy and safety of nintedanib in phase 2 trials and showed that a higher percentage of patients receiving nintedanib 150 mg twice daily experienced myocardial infarctions, the incidences of which were 1.5 and 0.4% in the nintedanib group and the placebo group, respectively (Richeldi et al., 2014).

## FDA-Approved SMKIs in 2017–2019

Since SMKIs in this group are relatively newly approved drugs, there is scarce information about drug-induced cardiotoxicity. The data of the cardiac AEs below were mainly collected from FDA-published prescribing information of these drugs.

### Midostaurin

Midostaurin is a multitargeted PKs inhibitor that inhibits FMS-related tyrosine kinase 3 (FLT3), KIT, PDGFRα/β, VEGFR2, and PKC. Midostaurin, in combination with chemotherapy, was the only drug approved by the FDA for the treatment of adult patients with newly diagnosed FLT3 mutation-positive acute myeloid leukemia (AML). The prescribing information from the FDA for midostaurin described that the midostaurin-induced cardiac AEs mainly included hypertension and pericardial effusion according to the safety evaluation of midostaurin-treated patients with FLT3 mutated AML at a dose of 50 mg twice daily (U.S. Food and Drug Administration, 2017e). The reported incidences of hypertension and pericardial effusion were 8 and 4%, respectively.

### Copanlisib

Copanlisib, a phosphatidylinositol-3-kinase (PI3K) inhibitor, was approved as an orphan drug for the treatment of adult patients with relapsed follicular lymphoma (FL). Hypertension was reported in the prescribing information of copanlisib (U.S. Food and Drug Administration, 2017a). According to the information, the clinical trials enrolled 168 patients treated with copanlisib at a dose of 60 mg on an intermittent schedule reported that all-grade hypertension occurred in 35% of patients, and grade 3 hypertension occurred in 26% of patients. Hence, blood pressure and cardiovascular disorders should be carefully monitored before and after each copanlisib infusion.

### Acalabrutinib

Acalabrutinib is a highly selective, covalent BTK inhibitor; and the drug was approved by the FDA as a second-line treatment of adult patients with MCL who have received prior therapies. As described in the prescribing information, cardiac AEs include AF or flutter and hypertension (U.S. Food and Drug Administration, 2017c). In 1,029 patients treated with acalabrutinib at a dose of 100 mg twice daily, the incidences of all-grade and grade 3 AF or flutter was 4.1 and 1.1%, respectively. Of note, the risk of acalabrutinib-induced cardiotoxicity might be increased in patients with pre-existing hypertension and arrhythmias.

### Fostamatinib

Spleen tyrosine kinase (SYK) is a main target of fostamatinib. The FDA approved fostamatinib as a second-line therapy for the treatment of thrombocytopenia in adult patients with chronic immune thrombocytopenia whose previous treatment is ineffective. According to the prescribing information, hypertension, which warns an increased cardiovascular risk was reported in 1% of patients with fostamatinib treatment at a dose of 100 mg twice daily; of note, patients with pre-existing hypertension might be more sensitive to the effects of fostamatinib-induced hypertension (U.S. Food and Drug Administration, 2018b).

### Gilteritinib

Gilteritinib is a potent TKI that inhibits FLT3 and AXL. The FDA approved gilteritinib for the treatment of relapsed or refractory AML with an FLT3 mutation in adult patients. The prescribing information of the FDA suggested that gilteritinib can induce QT interval prolongation (U.S. Food and Drug Administration, 2018c). As stated in the information, in clinical trials, 7% of gilteritinib-treated patients at the starting dose of 120 mg once daily experienced a QTc interval prolongation of greater than 60 ms from baseline and 1.4% experienced a QTc interval prolongation of greater than 500 ms.

### Zanubrutinib

Zanubrutinib inhibits BTK autophosphorylation and potently inhibits cell proliferation. The FDA approved zanubrutinib as a second-line therapy for the treatment of adult patients with MCL who failed in prior therapies. According to the prescribing information, treatment-related cardiac arrhythmias was reported as one of common cardiac AEs in patients treated with zanubrutinib monotherapy at the recommended dose of 160 mg twice daily or 320 mg once daily (U.S. Food and Drug Administration, 2019b). The incidence of cardiac arrhythmias was 2% and grade 3 or higher was 0.6%. Of note, patients with hypertension, acute infections or other cardiac risk factors may be at increased risk of zanubrutinib-induced cardiotoxicity.

### Fedratinib

Fedratinib is a selective inhibitor of Janus kinase 2 (JAK2) that promotes the growth and proliferation of cells. The major therapeutic indication for fedratinib is in the treatment of intermediate-2 or high-risk primary or secondary myelofibrosis in adults. As described in the prescribing information, several serious cardiac adverse reactions were reported. It was showed that cardiac failure occurred in 5% of patients receiving fedratinib 400 mg daily, and fatal adverse reactions of cardiogenic shock occurred in 1% of patients receiving fedratinib 400 mg daily (U.S. Food and Drug Administration, 2019c).

### Entrectinib

Entrectinib targets all kinds of NTRK fusions and targets ALK and ROS1 as well. The prescribing information of entrectinib stated that the QTcF interval was reported in patients across the entrectinib clinical trials including 355 patients who received entrectinib ranging from 200 to 600 mg once daily (U.S. Food and Drug Administration, 2019d). Among them, 3.1 and 0.6% of patients suffer from greater than 60 ms or 500 ms QTcF interval prolongation, respectively.

### Erdafitinib

Erdafitinib is the first approval of targeted agent for metastatic bladder cancer. It was indicated for the treatment of adult patients with locally advanced or metastatic urothelial carcinoma with an FGFR3 or FGFR2 alteration that has progressed on chemotherapy. As described in the prescribing information of erdafitinib, drug safety evaluated in the BLC2001 study enrolled 87 patients receiving erdafitinib 8 mg once daily showed that 1% of patients experienced acute myocardial infarction with a fatal outcome (U.S. Food and Drug Administration, 2019a).

## The Mechanisms of SMKIs-Induced Caridotoxicity

Through an analysis and comparison of reported cardiotoxic SMKIs, we recognized that some drugs for the same indications would indeed elicit similar types of cardiac adverse reactions. These findings suggested that some toxicities might arise due to concomitant inhibition of therapeutic targets in cardiomyocytes. The molecular mechanisms underlying SMKIs-induced cardiotoxicity are complex. However, SMKIs with similar targets bear a resemblance to the cardiotoxicity. It is reported that imatinib and ponatinib both targeting ABL revealed target-dependent cardiotoxicity. Studies have indicated that inhibition of ABL activity triggers cardiomyocyte endoplasmic reticulum (ER) stress and ROS excessive accumulation, which eventually result in cardiotoxicity caused by mitochondrial dysfunction (Force et al., 2007; Lamore et al., 2020). Some studies have reported that the structural changes of mitochondria with a decomposition trend are observed in imatinib-treated patients (Kerkela et al., 2006). Imatinib-induced ER stress, which leads to mitochondrial dysfunction is both observed *in vivo* and *in vitro* models. It can be observed that ponatinib can cause the accumulation of ROS and destroy the normal function of mitochondria In Human Induced Pluripotent Stem Cells (hiPSC)-induced cardiomyocytes (Talbert et al., 2015). VEGFs are closely related to the function of vascular endothelial cells and the maintenance of homeostasis. VEGF inhibitors are very likely to be associated with the risk of hypertension, such as sorafenib, sunitinib, axitinib, cabozantinib, copanlisib and so on (Li et al., 2015). HER2 is very important target which is responsible for the maintenance of cardiac contractile. It has been reported that SMKIs targeting HER2, lapatinib and osimertinib, for example, may lead to a decrease of intracellular ATP production and activation of apoptosis signalling pathways, which bring about cardiotoxicity (Grazette et al., 2004). In future drug development, we can determine a rational approach to prophylaxis according to these reported functional kinase targets.

A reduction in the amount of cardiomyocytes is one of the main phenotypes of cardiotoxicity. Deeper mechanical research indicated that SMKIs not only contribute to compromised cardiac function but also directly lead to ongoing cell apoptosis, autophagy, and necrosis (Lamore et al., 2020). The cardiotoxicity induced by SMKIs is usually *via* multiple pathways with cross-links. Apoptosis caused by SMKIs can be observed in many *in vitro* models of cardiotoxicity. For example, apoptosis was both observed in mouse and rat cardiomyocytes under the treatment of the multi-target inhibitor sunitinib. Kerkela et al. (2006) found that the apoptotic morphology changed in isolated rat primary cardiomyocytes after imatinib treatment. Moreover, autophagy is involved in regulating the metabolic homeostasis of cardiomyocytes. Zhao et al. (2010) proposed that autophagy was involved in the occurrence of sunitinib-induced cardiotoxicity for the first time. Subsequently, some researchers found that imatinib, sorafenib and lapatinib treated heart tissue could occur the obvious increase of autophagosomes and autophagolysosomes, accompanied by a decrease in myocardial contractility (Jacob et al., 2016). In addition to that two cell death pathways involved in the process of cardiotoxicity, our research team confirmed a relative new pathway, programmed necrosis, was involved in dasatinib-induced cardiotoxicity *in vitro* (Xu et al., 2018). Hence, there is an urgent need to understand the potential mechanism of SMKIs and develop available strategies to resolve cardiac side effects.

## Conclusion and Discussion

**Figure 1:**
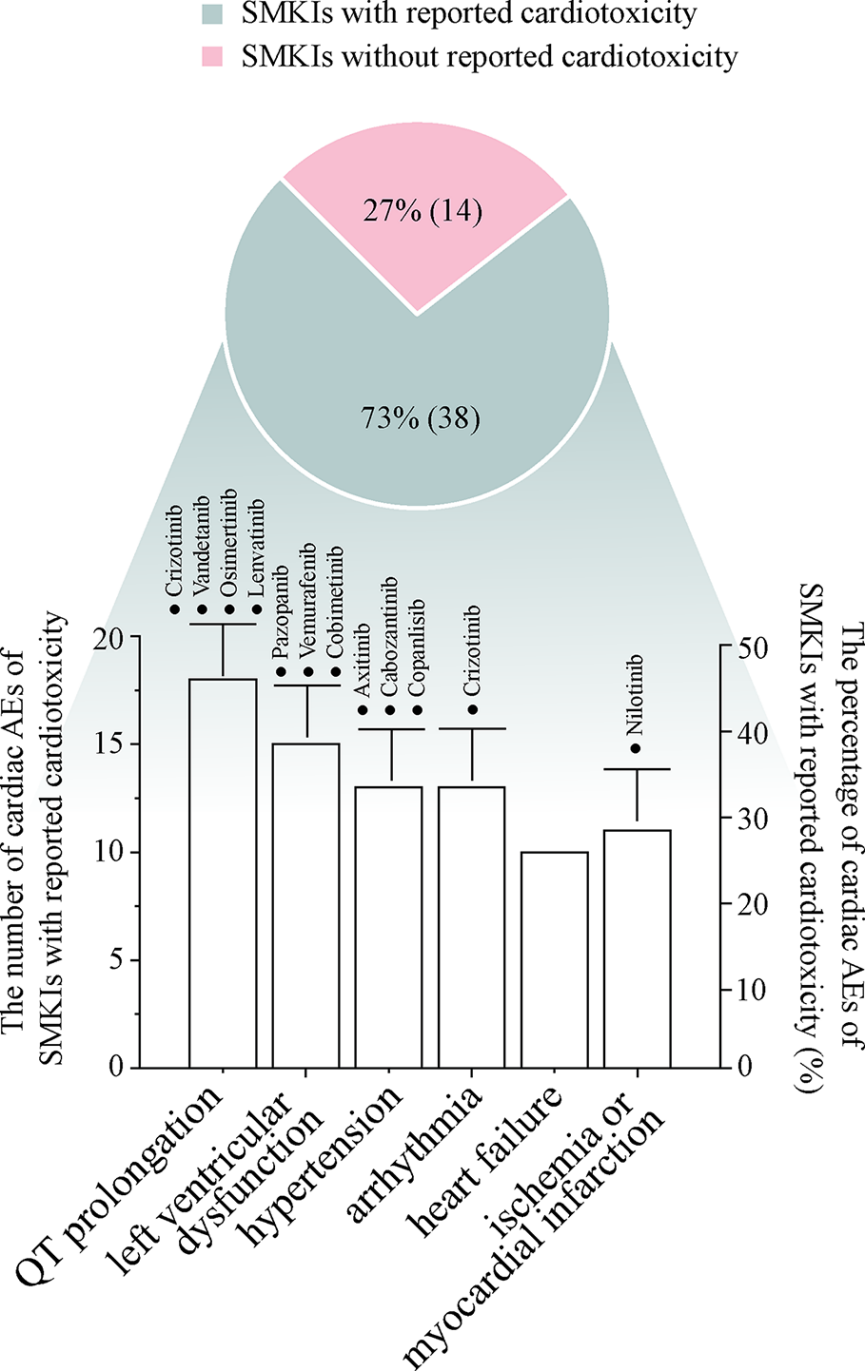
The pie diagram shows the percentage of SMKIs with reported cardiotoxicity in 52 FDA-approved SMKIs, and the bar chart describes the numbers and percentages of representative cardiac adverse events in 38 SMKIs with cardiotoxicity. The drugs with ≥10% incidence of cardiac adverse events are highlighted in the bar chart.

Research has shown that SMKIs therapy can change malignant neoplasm to a manageable chronic disease; therefore, a large proportion of patients receive long-term SMKIs therapy. In this review, we have outlined the occurrence from 42 clinical trials, 25 FDA-published documents, seven meta-analysis/systematic reviews and three case reports associated with the cardiotoxicity of 52 FDA-approved SMKIs. To date (**Figure 1**), 73% (38 of 52) of SMKIs have reported treatment-related cardiotoxicity. Among the 38 SMKIs with known cardiotoxicity, the incidences of cardiac adverse events were QT prolongation: 47% (18 of 38), hypertension: 40% (15 of 38), LV dysfunction: 34% (13 of 38), arrhythmia: 34% (13 of 38), HF: 26% (10 of 38) and ischemia or myocardial infarction: 29% (11 of 38). Most of the drugs were at FDA-recommended doses initially in clinical trials. Severe cardiac AEs in SMKI-treated patients usually lead to a dose reduction or dose interruption, which is harmful to prolongation of survival time in patients. Moreover, we surveyed clinical reports and found that in a few cases, patients died from cardiotoxic side effects of SMKIs, giving oncologists and cardiologists a wake-up call. Collecting and understanding comprehensive information on SMKI-induced cardiotoxicity are of utmost importance to promote life quality and prolong survival time of patients.

In the development process of novel SMKIs, we should pay more attention to balancing the treatment efficacy and the risk of cardiotoxicity. In preclinical drug studies, producing an accurate, reliable and easy-going cardiotoxicity evaluation model is of key importance. Further development and refinement of models such as stem cell-derived human cardiomyocytes, zebrafish, and rodent models in drug toxicity screening enriching the means of cardiac assessment are in urgent need. During clinical drug application, we should develop a global standard for echocardiography monitoring and search for more sensitive and stable serum biomarkers of heart injury in a timely and appropriate manner to intervene in the development of cardiac-related complications.

The data of this review are mainly collected from clinical trials, which lack preclinical studies including *in vivo* or *in vitro* studies. The consistency of SMKI-induced cardiotoxicity in clinical studies and preclinical studies remains unclear. The conclusion of this review is time-sensitive. In the future, more drug associated cardiac AEs might be reported with the large-scale clinical application of SMKIs, especially some recently approved drugs. It is of vital importance to carefully monitor cardiac AEs occurred in patients receiving SMKIs. In addition, the discussion regarding the molecular mechanisms underlying the SMKI-induced cardiotoxicity is incomplete. Further studies are needed to clarify the mechanisms of SMKI-induced cardiotoxicity. We found that scarce information of and relative low incidences of cardiotoxicity were reported in newly approved SMKIs in the past three years. On one hand, drug safety of new-generation SMKIs with optimized structures ought to better than that of the old drugs. On the other hand, the occurrence of some cardiac AEs has not been recorded comprehensively due to insufficient clinical patients, which needs to be followed up and updated in the future.

## Author Contributions

XY and PL conceived the review article and made the corrections in the manuscript. QH provided some critical comments. YJ wrote the manuscript. ZX and HY collected the related research articles.

## Funding

This study was supported by the National Natural Science Foundation of China (81872941 and 81673522) and Science Technology Plan Project of Zhejiang Province (2019C04010).

## Conflict of Interest

The authors declare that the research was conducted in the absence of any commercial or financial relationships that could be construed as a potential conflict of interest.

## Footnotes

1 The prescribing information of SMKIs in this review is available at: www.fda.gov.
